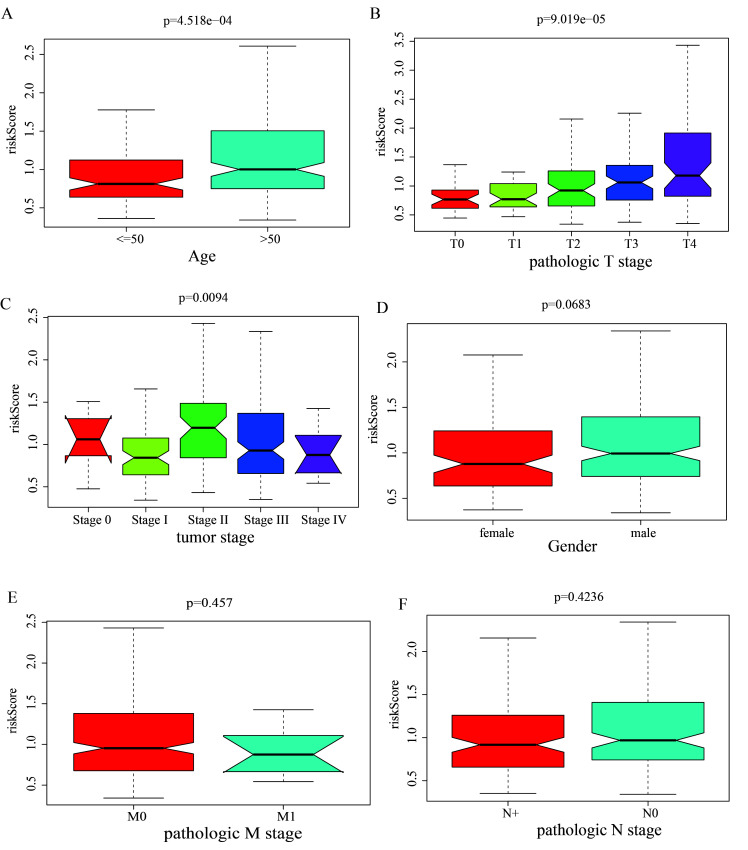# Identification of a metabolism-linked genomic signature for prognosis and immunotherapeutic efficiency in metastatic skin cutaneous melanoma

**DOI:** 10.1097/MD.0000000000038347

**Published:** 2024-06-07

**Authors:** Zhongshun He, Jing Lyu, Lechun Lyu, Xiaolin Long, Biao Xu

**Affiliations:** a Department of Oral and Maxillofacial Surgery, Kunming Medical University School and Hospital of Stomatology, Kunming, China; b Yunnan Key Laboratory of Stomatology, Kunming, China; c Department of Physiology, Kunming Medical University, Kunming, Yunnan, China; d Technology Transfer Center, Kunming Medical University, Kunming, Yunnan, China; e Yunnan Bestai Biotechnology Co., Ltd., Kunming, Yunnan, China.

**Keywords:** immunotherapy, metabolism-linked genes, metastatic skin cutaneous melanoma, nomogram, prognosis, tumor micro-environment

## Abstract

Metastatic skin cutaneous melanoma (MSCM) is the most rapidly progressing/invasive skin-based malignancy, with median survival rates of about 12 months. It appears that metabolic disorders accelerate disease progression. However, correlations between metabolism-linked genes (MRGs) and prognosis in MSCM are unclear, and potential mechanisms explaining the correlation are unknown. The Cancer Genome Atlas (TCGA) was utilized as a training set to develop a genomic signature based on the differentially expressed MRGs (DE-MRGs) between primary skin cutaneous melanoma (PSCM) and MSCM. The Gene Expression Omnibus (GEO) was utilized as a validation set to verify the effectiveness of genomic signature. In addition, a nomogram was established to predict overall survival based on genomic signature and other clinic-based characteristics. Moreover, this study investigated the correlations between genomic signature and tumor micro-environment (TME). This study established a genomic signature consisting of 3 genes (*CD38, DHRS3*, and *TYRP1*) and classified MSCM patients into low and high-risk cohorts based on the median risk scores of MSCM cases. It was discovered that cases in the high-risk cohort had significantly lower survival than cases in the low-risk cohort across all sets. Furthermore, a nomogram containing this genomic signature and clinic-based parameters was developed and demonstrated high efficiency in predicting MSCM case survival times. Interestingly, Gene Set Variation Analysis results indicated that the genomic signature was involved in immune-related physiological processes. In addition, this study discovered that risk scoring was negatively correlated with immune-based cellular infiltrations in the TME and critical immune-based checkpoint expression profiles, indicating that favorable prognosis may be influenced in part by immunologically protective micro-environments. A novel 3-genomic signature was found to be reliable for predicting MSCM outcomes and may facilitate personalized immunotherapy.

## 1. Introduction

Metastatic skin cutaneous melanoma (MSCM) is a highly invasive form of skin malignancy.^[1]^ MSCM incidence and mortality rates have steadily increased over the last several decades.^[2,3]^ The high invasiveness and metastatic ability of MSCM cells cause rapid progression and frequent recurrence and resulting in poor prognoses for many MSCM cases, with long-term survival rates of <1%.^[4,5]^ Fortunately, recent advancements in targeted therapies and immunotherapies have increased case survival.^[6–8]^ However, most cases remain prone to drug resistance and adverse reactions, severely limiting treatment options.^[9,10]^ In order to optimize MSCM treatment strategies, it is necessary to further elucidate the molecular differences between primary skin cutaneous melanoma (PSCM) and MSCM and to identify novel and effective prognostic and therapeutic biomarkers.

Recent studies have provided strong evidence that cancer metastasis is intimately related to metabolism.^[11–15]^ In the case of endometrial tumors, suppression of phosphoglucose isomerase (PGI), a glycolytic enzyme secreted by cancer cells, can affect epithelial-mesenchymal transition.^[16]^ Glyceraldehyde-3-phosphate dehydrogenase (GAPDH) silencing inhibits epithelial-mesenchymal transition in colon tumors by downregulating SNAIL.^[12]^ In addition, previous studies have demonstrated that FABP5 can exacerbate lymph-node metastases by reprograming fatty acid metabolism in cervical cancer.^[17]^ Glutaminolysis disorders can also regulate in vivo metastasis by targeting GLS1.^[15]^ More importantly, abnormal lipid metabolism can act as a crucial secondary messenger for shifting the tumor micro-environment (TME), resulting in tumor progression.^[18–21]^ Several studies, however, have shown that metabolic reprogramming is closely linked to MSCM or PSCM. For example, metabolic differences among melanoma cells caused by MCT1 transporter function differences can result in differences in metastatic ability.^[22]^ In addition, emerging investigations have highlighted that tumor oxidative metabolism is a potential target-pathway for enhancing immune-based-therapeutic responses.^[23]^ On the other hand, it has been proposed that metabolic shifts/interactions between tumor cells and the micro-environment within skin cutaneous melanoma can affect melanoma development/immune-based responses.^[24]^ In addition, prior research has revealed that nicotinamide phosphoribosyltransferase plays a crucial role in MSCM by targeting metabolic reprogramming.^[25]^ However, it is unknown how metabolic disorders affect the progression of MSCM.

TME, which consists of blood vessels, infiltrated immune cells, fibroblasts, and other stromal cells, has been discovered to play a crucial role in tumor development.^[26]^ Furthermore, increasing evidence indicates that TME-related factors have a significant impact on tumor development and therapeutic responses.^[27,28]^ TME homeostasis can be regulated by metabolic processes, which provide TME with energy and metabolites. For example, cancer-associated fibroblasts and adipocytes can transport nutrients, such as alanine/lipids, to the TME to maintain malignant cell growth.^[29,30]^ In addition, cancer cells can acquire immune-based cellular functions through catabolites such as lactate.^[31]^ In addition, one study suggests that inhibiting PD-1 or PD-L1 enhances glycolytic activity of T-cells by increasing glucose presence in the TME,^[32]^ whereas another study demonstrates that CTLA-4 signaling inhibition can reduce AKT phosphorylation and activation,^[33]^ which may lessen the enhanced glucose metabolism/ mitochondrial remodeling that occurs after T-cell activation. Therefore, metabolic imbalance may cause the changes of TME, ultimately leading to tumor development and influencing immunotherapy in clinical practice.

Recent evidence demonstrates that metabolism-linked genes (MRGs) could be selected as prognosis-based biomarkers for cancers, such as skin cutaneous melanoma.^[34–36]^ Mou et al proposed that iron MRGs could be used to determine prognosis in cases of clear-cell renal-cell carcinoma.^[34]^ Furthermore, Wu et al discovered that lipid MRGs could predict the survival of diffuse gliomas.^[35]^ In addition, Zeng et al formed a prognosis-based model for surviving skin cutaneous melanoma based on MRGs.^[36]^ However, additional research is required to determine whether MRGs can be used as prognosis-based biomarkers for MSCM. Thus, for this investigation, gene expression data obtained from The Cancer Genome Atlas (TCGA) database (https://tcga-data.nci.nih.gov/tcga/) and Gene Expression Omnibus (GEO) database, (https://www.ncbi.nlm.nih.gov/geo/) were employed for constructing and verifying a genomic signature based on MRGs. In addition, this study investigated potential links between the linked genomic signature of MRGs and the micro-environment of MSCM, as well as whether the linked genomic signature of MRGs could be used to guide immunotherapy selection in MSCM cases, which could contribute to the therapeutic options available for MSCM cases.

## 2. Materials and methods

### 2.1. Data collection

TCGA was utilized to collect data sets containing mRNA expression information from 103 PSCM cases and 365 MSCM cases, as well as complementary medical data. Furthermore, the GSE65904 dataset was extracted from the GEO database and contains survival information for 16 PSCM cases and 198 MSCM cases. MRGs were collected from the MsigDB (Table S1, Supplemental Digital Content, http://links.lww.com/MD/M676).

### 2.2. Identification of DEGs and differentially expressed metabolism-linked genes

The samples were organized into cohorts based on the PSCM/MSCM cases. The Limma package in R was employed to assess genomic expression.^[37]^ A log_2_| fold-change| >0.5/adjust *P < *.05 were deemed to confer a statistically significant variation upon DEG screens. The TCGA database produced 4499 differentially expressed genes (DEGs), while 589 DEGs were screened from the GSE65904 dataset (Table S2, Supplemental Digital Content, http://links.lww.com/MD/M677 and S3, http://links.lww.com/MD/M678). The genes in both TCGA-DEGs, GSE-DEGs, and MSigDB-MRGs were deemed to be differentially expressed MRGs (DE-MRGs).

### 2.3. Establishment of a prognosis-based genomic signature

The TCGA-MSCM dataset was utilized to identify DE-MRGs associated with overall survival (OS). Analysis of univariate Cox regression was used to identify prognosis-based DE-MRGs, with genes considered significant at a *P < .05* cutoff. LASSO-penalized Cox regression analyses were conducted to increase the selection of OS prognosis genes in MSCM cases.^[38]^ As a result, a multivariate Cox regression model was utilized to develop a prognosis-based classifier by selecting genes across these prognosis-based DE-MRGs. Risk scoring, based on the expression profiles of individual cases, was quantified using the Cox regression model. Risk scoring was generated as follows:


$$\begin{array}{l} Risk\;score=\frac{e^{sum\left(each\;gene^{\prime }s\;expression\;levels\;\times \;corresponding\;coefficient\right)}}{e^{sum\left(each\;gene^{\prime }s\;mean\;expression\;levels\;\times \;corresponding\;coefficient\right)}} \end{array}$$

Cases were divided into low and high-risk cohorts based on the median risk score. The R package “survivalROC” was utilized to generate a time-dependent ROC curve for assessing the predictive value of this prognosis-based genomic signature in OS.^[39]^ Using the “survival” R package, a K-M survival curve was employed to compare survival variations between high- and low-risk cohorts. Furthermore, stratified survival analyses were carried out across cases having differing age brackets (younger, older), gender (male, female), pathologic T-stage (T_1_-T_2_, T_3_-T_4_), pathologic N-stage (N^+^, N_0_), pathologic M-stage (M_0_, M_1_), and tumor-stage (stage I/II, stage III/IV). Univariate/ multivariate survival assessments were carried out through Cox regression model, while a nomogram plot was developed based on the Cox regression coefficients.

### 2.4. Validation of the prognosis-based model in the gene expression omnibus database

The prognosis-based model was validated using the GSE65904 dataset. Only cases with clear information on survival, age, gender, pathologic N-stage, pathologic T-stage, tumor-stage, and pathologic M-stage were included in the study. Finally, 135 cases were added from the GSE65904 dataset to the validation set.

### 2.5. Functional/annotation analyses

The Hallmark gene sets, which were also obtained from the Broad Institute’s MSigDB,^[40]^ were used to examine changes in pathway enrichment based on a prognosis-based model using the R package “Gene Set Variation Analysis (GSVA).”^[41]^ Markedly enriched pathways in Hallmark gene sets were deemed at *P < *.05.

### 2.6. ESTIMATE algorithm

Through transcriptomic expression profiling of tumor samples, the ESTIMATE algorithm can identify infiltrations of immune-based/stromal cells.^[42]^ Genomic expression values were graded/ranked across individual samples. Combining variations across functions of empirical cumulative distribution for the signature gene and the remaining genes led to the determination of the statistical significance value. Through ssGSEA, the ESTIMATE algorithm provided stromal/immune-based scorings (43).

### 2.7. Immune-based infiltrates in high/low-risk cohorts

The ssGSEA method^[43]^ and the MCP-counter method^[44]^ quantified tumor-infiltrating immune-based cellular levels, depending upon high-/low-risk cohorts. The ssGSEA marker genes were present in 28 cell types based on the immune system. “pheatmap” R package was used to generate the figures.

### 2.8. Statistical analysis

R® v3.4.3 was used for statistical analysis. Unless otherwise specified*, P < .05* was considered to confer statistical significance.

### 2.9. Ethics approval statement

This study is a bioinformatics research paper and does not involve experiments with animals or human subjects. The study does not need to be approved by moral and ethical clerks.

## 3. Results

### 3.1. Differentially expressed metabolism-linked gene screening across public databases

**Figure 1.**
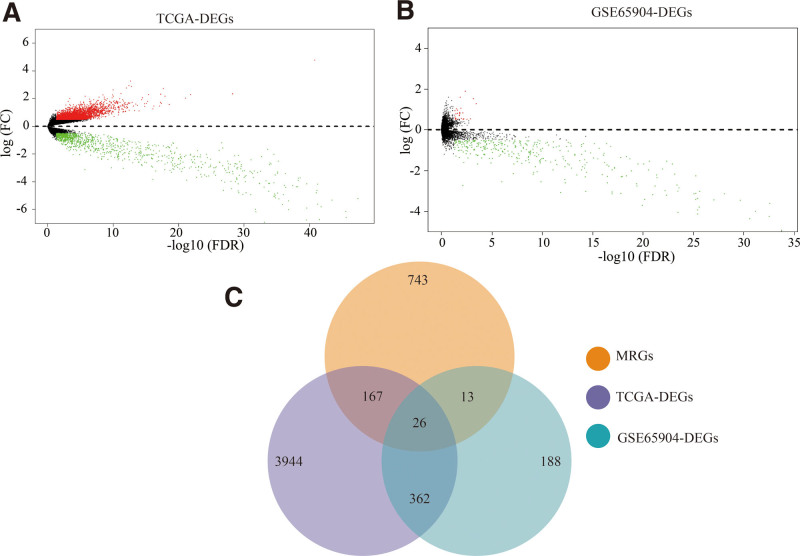
Screening of DE-MRGs from TCGA and GEO databases. (A) Volcano plot of the DEGs in MSCM samples compared to PSCM tissues based on the TCGA database. (B) Volcano plot of the DEGs in MSCM samples compared to PSCM tissues based on the GSE65904 data set. (C) Overlapping genes among DEGs in TCGA, DEGs in GSE65904, and MRGs. DEGs = differentially expressed genes, DE-MRGs = differentially expressed MRGs, GEO = Gene Expression Omnibus, MRGs = metabolism-linked genes, MSCM = metastatic skin cutaneous melanoma, PSCM = primary skin cutaneous melanoma, TCGA = the cancer genome atlas.

TCGA/GEO was used to collect genomic data. According to Limma analysis results, a total of 4499 DEGs were identified in MSCM tissues compared to PSCM tissues in the TCGA database, and 589 DEGs were identified in MSCM samples compared to PSCM samples in the GEO database. Figure 1A and B depict the volcano plot for individual genomic expression profile results. Tables 1 and 2 show the top ten up- and down-regulated DEGs from the TCGA and GEO databases, respectively. The MSigDB database identified 949 metabolism-related genes (MRGs). The results of the GEO, TCGA, and MSigDB analyses revealed 26 overlapping genes, which were labeled as DE-MRGs (Fig. 1C).

**Table 1:** The top 10 up- and down-regulated DEGs in the TCGA database.

Gene	logFC	AveExpr	t	*P* value	Adj. *P* value	B
C7	4.789509874	8.006581216	15.66284062	8.59E-45	1.63E-41	90.99608513
LINC01235	2.3494763	5.386290523	12.53416105	2.71E-31	5.84E-29	60.28664617
DNAJC5B	2.309311295	4.738948767	10.86039043	1.15E-24	1.51E-22	45.23014379
ADAMTSL3	2.042321642	6.126470587	10.66492861	6.30E-24	8.04E-22	43.55253169
CD1D	1.588853767	6.475207891	9.944853299	2.83E-21	2.87E-19	37.53621553
CR1	2.191392421	6.364760466	9.768597717	1.21E-20	1.18E-18	36.10477001
SHISA3	2.724245588	3.29841543	9.696106481	2.19E-20	2.10E-18	35.52093368
SLC8A1	1.658180426	7.973006071	9.598788754	4.84E-20	4.54E-18	34.74169773
TNFSF11	2.33172698	4.392452161	9.575289463	5.85E-20	5.46E-18	34.55432367
EBF2	1.731004726	5.918051749	9.559545164	6.65E-20	6.18E-18	34.42895724
S100A7A	−5.206469128	1.938186523	−17.3549418	1.70E-52	3.57E-48	108.5243597
S100A7	−6.585336511	3.48056173	−16.89331841	2.25E-50	2.36E-46	103.6978861
KRT17	−6.12499924	6.850129119	−16.83652788	4.10E-50	2.86E-46	103.1061823
KLK5	−5.316007777	2.389359965	−16.34774104	6.94E-48	3.63E-44	98.03406231
CASP14	−5.788473496	2.52260874	−15.96101814	3.91E-46	1.64E-42	94.04945456
KRT6B	−6.721546588	5.113369052	−15.88741533	8.39E-46	2.93E-42	93.29416565
WFDC5	−4.154826043	1.568865548	−15.80132849	2.05E-45	5.92E-42	92.4120759
KRTDAP	−5.61013043	3.184620722	−15.79180871	2.26E-45	5.92E-42	92.3146191
KRT75	−4.609870959	2.022698665	−15.7618898	3.08E-45	7.17E-42	92.00844498
LGALS7	−4.771404747	2.260199843	−15.72958771	4.31E-45	9.01E-42	91.67807926

DEGs = differentially expressed genes.

**Table 2:** The top 10 up- and down-regulated DEGs in the GSE65904 data set.

Gene	logFC	AveExpr	T	*P* value	Adj. *P* value	B
RARRES1	1.278580041	9.31785577	4.732339369	4.04E-06	0.000366553	3.836043645
APOC1	1.521534723	11.8352411	4.562199646	8.53E-06	0.000718728	3.126627537
NHLRC3	0.5299981	8.99154001	4.400173547	1.71E-05	0.001382217	2.470884865
EIF4A2	0.506517929	13.1405198	4.186457427	4.14E-05	0.003076437	1.636234871
C7	1.881419752	9.210694966	4.07466732	6.50E-05	0.00461805	1.213645504
CLK1	0.522337851	9.315159807	3.985755967	9.23E-05	0.006291871	0.884518171
PSIP1	0.50831096	8.839101698	3.940196736	0.000110305	0.007309684	0.718286729
HOXB2	0.827706571	8.421686049	3.924377702	0.000117284	0.007655432	0.660953262
FMNL2	0.54510748	8.30357798	3.81204058	0.000180339	0.011085384	0.259554934
ALDH1A1	1.058823114	10.32954818	3.788993881	0.000196747	0.012046814	0.178458224
KRT6A	−4.937628437	7.313145515	−16.18012806	5.83E-39	1.83E-34	77.16667138
KRT6B	−3.615909946	7.234148697	−15.71821833	1.70E-37	2.67E-33	73.89952271
KRT17	−4.257588623	7.319722649	−15.6439422	2.93E-37	3.07E-33	73.37312652
KRT6C	−4.00060288	7.098675718	−15.17991834	8.77E-36	6.89E-32	70.07924972
S100A7	−4.368393304	7.259112527	−14.96729957	4.17E-35	2.62E-31	68.56742788
MGC102966	−3.452539634	7.230550146	−14.64502448	4.44E-34	2.32E-30	66.27373308
LOC400578	−3.15094235	7.164963413	−14.13212146	1.91E-32	8.58E-29	62.62042786
KRT16	−3.428009333	7.148226632	−13.78516281	2.44E-31	9.56E-28	60.14922201
LOC729252	−3.022217193	7.165673863	−13.33034773	6.81E-30	2.38E-26	56.91306342
PI3	−3.802943476	7.37213807	−13.29727082	8.67E-30	2.72E-26	56.67794779

DEGs = differentially expressed genes.

### 3.2. Development/validation for prognosis-based modeling, depending upon differentially expressed metabolism-linked genes

**Figure 2.**
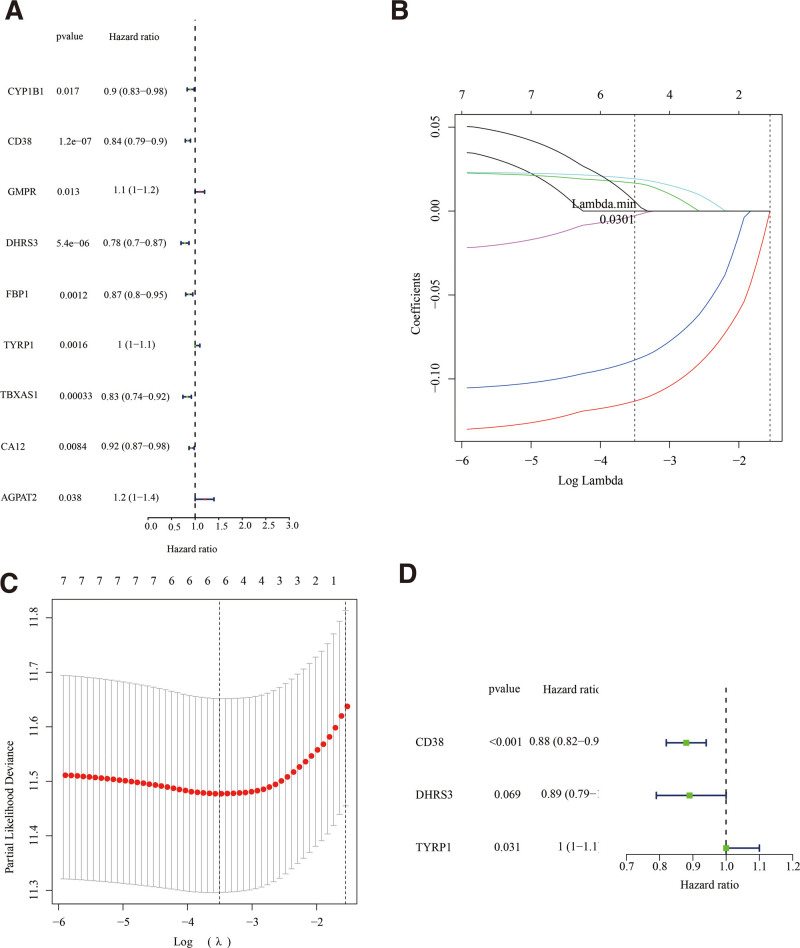
Regression analysis in TCGA databases to generate the genomic signature based on DE-MRGs. (A) Forest map of prognosis-based DE-MRGs by univariate Cox regression analysis. (B) LASSO coefficient spectrum of 6 genes. (C) The most proper log (Lambda) value in the LASSO model. (D) 3 genes (CD38, DHRS3, and TYRP1) were selected to construct a genomic signature by multivariate Cox regression analysis. DE-MRGs = differentially expressed MRGs, TCGA = the cancer genome atlas.

9 of the 26 DE-MRGs were associated with prognoses, as confirmed by univariate Cox regression assessments (Fig. 2A; Table S4, Supplemental Digital Content, http://links.lww.com/MD/M679). Six DE-MRGs, *CD38, GMPR, DHRS3, TYRP1, CA12*, and *AGPAT2*, were chosen based on their peak-optimized potential prognostic significance, as determined by LASSO regression analyses (Fig. 2B and 2C; Table S5, Supplemental Digital Content, http://links.lww.com/MD/M680). A K-M assessment concerning OS was employed to identify 6 DE-MRGs in order to obtain additional validation over optimal prognostic DE-MRGs. High *GMPR, TYRP1*, and *AGPAT2* gene expression was correlated with a poor prognosis, whereas down-regulated *CD38, DHRS3*, and *CA12* gene expression was correlated with a poor prognosis (Figure S1, Supplemental Digital Content, http://links.lww.com/MD/M697). In a multivariate cox regression, 3 variables, including *CD38, DHRS3*, and *TYRP1*, were selected for the development of a predictive model (Fig. 2D; Table S6, Supplemental Digital Content, http://links.lww.com/MD/M681).

**Figure 3.**
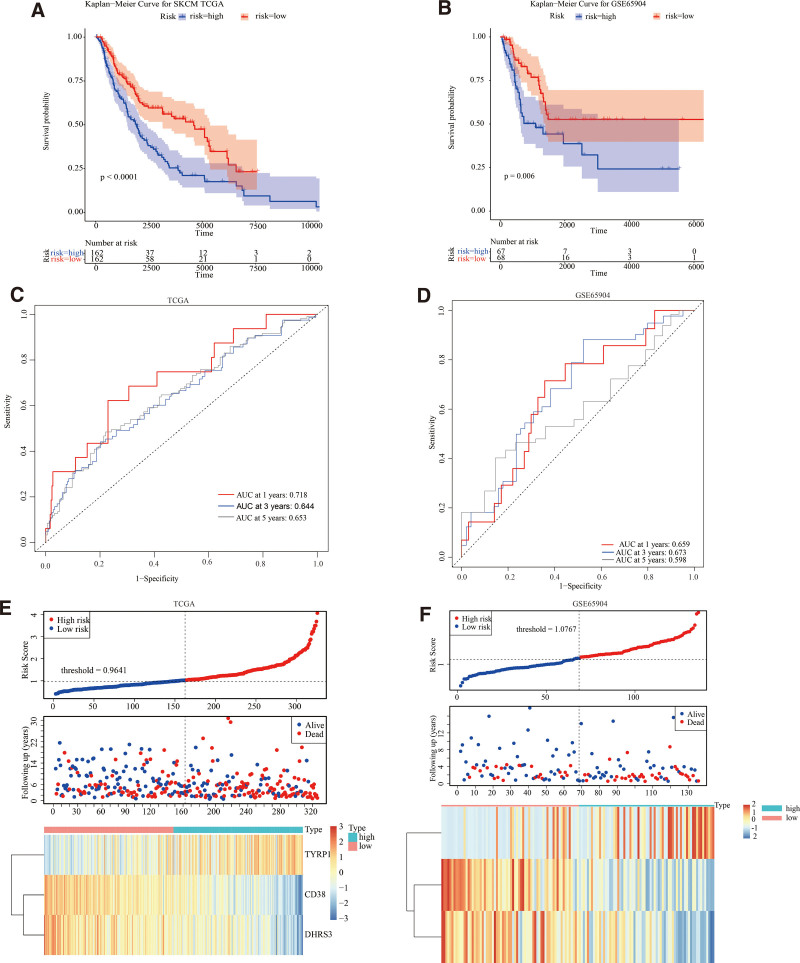
The assessment and validation of the efficiencies for genomic signature in the TCGA database and the GSE65904 data set. (A, B) Kaplan–Meier survival curves showed the prognosis-based value of the genomic signature in TCGA database (A) and GSE65904 data set (B). (C, D) ROC curves assessed the efficiency of the genomic signature for predicting 1-, 3- and 5-y survival in the TCGA database (C) and the GSE65904 data set (D). (E, F) The expression profiles of 3 genes, the distribution of risk scores, and the survival status of cases in the TCGA database. (E) and GSE65904 data set (F). TCGA = the cancer genome atlas.

Cases of MSCM were divided into low and high-risk cohorts according to the median risk score. High-risk cases had significantly worse OS (*P < *.0001) compared to low-risk cases (Fig. 3A). The distribution of genomic expression and risk scores is depicted in Figure 3E. *TYRP1* expression was upregulated in the high-risk cohort, whereas *CD38* and *DHRS3* expression was down-regulated. This study employed ROC analysis to determine the sensitivity and specificity of this prognosis-based model. The time-dependent area under the curves area under the curves for 1-, 3-, and 5-year OS rates for MSCM cases using a prognosis-based model were 0.718, 0.644, and 0.653, respectively (Fig. 3C). In addition, the prediction capability of this prognosis-based model was evaluated using 135 MSCM samples with survival status and OS-time from the validation dataset (GSE65904). Cases were separated into low and high-risk cohorts using the previously described formula, based on the validation dataset’s median risk score. Corroborating previous findings from this study, MSCM cases in the high-risk cohort component of the validation set had significantly lower median OS compared to those in the low-risk cohort (*P* = .006; Fig. 3B). Figure 3F depicts the risk scoring and genomic expression distribution. The time-dependent area under the curves for 1-, 3-, and 5-year OS rates in MSCM cases using a prognosis-based model were 0.659, 0.673, and 0.598, respectively (Fig. 3D).

### 3.3. Stratified survival analysis

**Figure 4.**
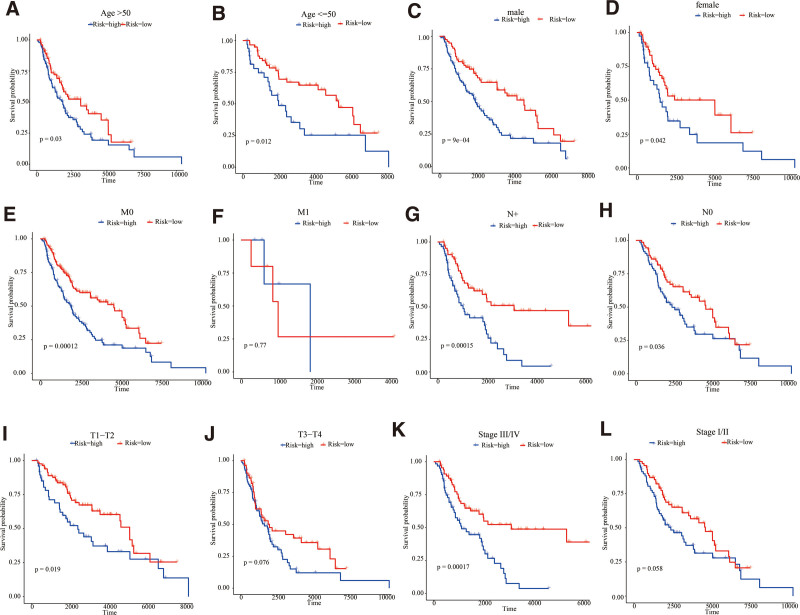
Kaplan–Meier survival stratification analyses in TCGA database. (A) Age > 50 yr. (B) Age < = 50 yr. (C) Male. (D) Female. (E) Pathology M0. (F) Pathology M1. (G) Pathology N+. (H) Pathology N0. (I) Pathology T1-T2. (J) T3-T4. (K) Stage I/II. (L) Stage III/IV. TCGA = the cancer genome atlas.

TCGA was used to collect clinic-pathological data from MSCM cases, such as age, gender, pathologic N-stage, pathologic T-stage, tumor-stage, and pathologic M-stage. Pathologic N-stage, pathologic T-stage, tumor-stage, and age were significantly correlated with MSCM case survival, as determined by K-M survival analyses (all *P < *.01; Figure S2, Supplemental Digital Content, http://links.lww.com/MD/M698). Associations between the prognosis-based model and clinic-based characteristics were evaluated in order to define prognosis-based model functions in MSCM development. The boxplot revealed that high-risk tended to be elderly, high pathologic T-stage and tumor-stage II cases, indicating a significant correlation between the prognosis-based model and tumor aggressiveness (Figure S3, Supplemental Digital Content, http://links.lww.com/MD/M699). If this prognosis-based model can be applied to various clinic-pathological profiles, stratification survival assessments will be evaluated. This study concluded that the prognosis-based model can effectively predict OS across nearly all subgroups with distinct clinical profiles (Fig. 4).

### 3.4. Establishment of a prognosis-based nomogram for OS prediction in metastatic skin cutaneous melanoma

**Figure 5.**
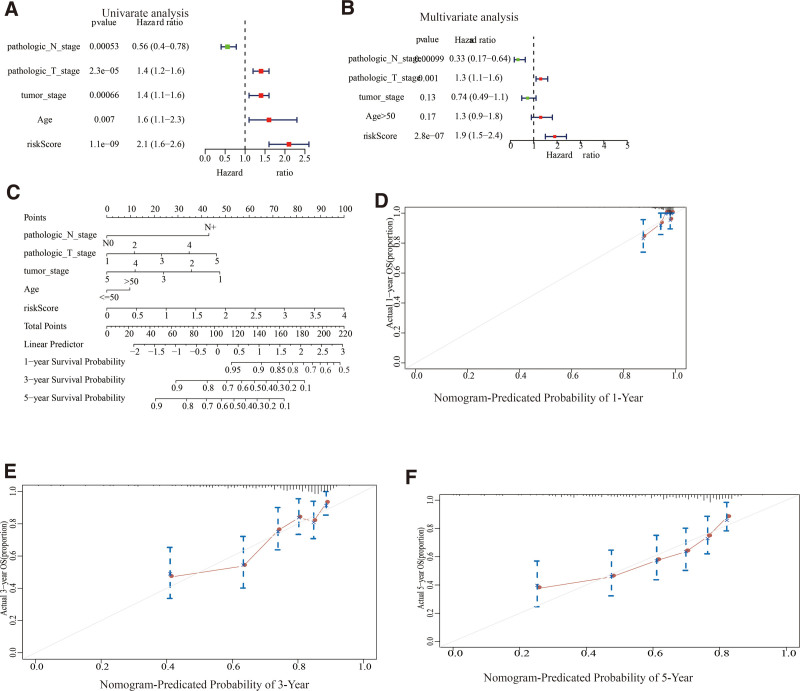
The development of a nomogram to predict the 1-, 3-, and 5-year MSCM survival in the TCGA database. (A, B) Forest plot summary of the univariate (A) and multivariable (B) Cox analyses of risk scores and clinicopathological features. (C) Nomogram based on risk scoring and clinicopathological characteristics for predicting MSCM survival at 1, 3, and 5 years. (D, E, F) Calibration curves of the nomogram for predicting the 1-, (D) 3- (E) and 5-year (F) survival of MSCM. MSCM = metastatic skin cutaneous melanoma, TCGA = the cancer genome atlas.

To determine whether this prognosis-based model was independent of clinic-pathological profiles, univariate and multivariate Cox regression analyses were conducted using risk scoring, tumor-stage, gender, age, and pathologic TNM stage as covariates (Table S7, Supplemental Digital Content, http://links.lww.com/MD/M682). This evaluation indicated that the prognosis-based model was a significant independent factor for OS (HR = 1.9, *P* = 2.8e-07; Fig. 5A and B). In order to provide clinicians with a quantitative method for predicting individuals with survival times of 1, 3, and 5 years, this study established a prognosis-based nomogram that incorporated clinic-pathological-independent risk factors with this prognosis-based model (Fig. 5C). The C-index for this nomogram was approximately 0.69. Furthermore, nomogram calibration curves demonstrated adequate agreements across predicted rates of 1-, 3-, and 5-year OS and actual monitoring outcomes (Fig. 5D, E and F).

### 3.5. Identification of the prognosis-based model-linked functional annotation

**Figure 6.**
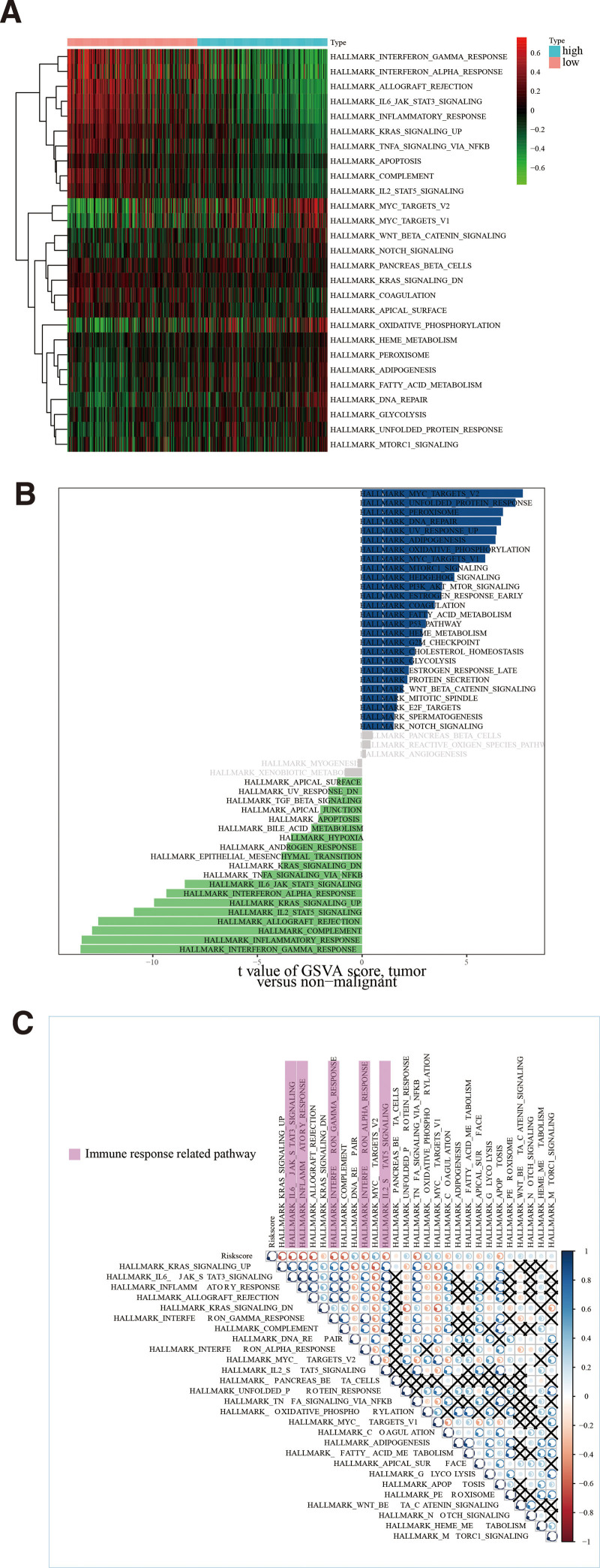
Functional annotation of the genomic signature in the TCGA database. (A) The hallmark gene sets’ cluster heat maps in high- and low-risk cohort cases. (B) The bar graph displayed the outcomes of GSVA. (C) The association between risk and enrichment scores for hallmark gene sets. GSVA = Gene Set Variation Analysis, TCGA = the cancer genome atlas.

The GSVA was employed to identify dynamics for biological pathways based on a prognosis-based model and Hallmark gene sets. Detailed dataset outcomes for GSVA are listed in Table S8, Supplemental Digital Content, http://links.lww.com/MD/M683. This study discovered that high-risk cohort genes were mostly enriched for metabolic/cancer-linked pathways, such as the P53 signal pathway, the P13k-Akt signal pathway, acid metabolism, and so on, all of which play important roles in tumorigenesis. Furthermore, metabolism-related gene sets were found to be overrepresented in the low-risk cohort (Fig. 6A and B). The Hallmark gene sets were subjected to correlation analysis in an effort to elucidate functional annotations comprehensively. Gene sets in immune-based response-linked pathways were significantly more interconnected than gene sets in other pathways, with risk scores being strongly inversely related to all immune-linked gene sets (Fig. 6C). These results demonstrated that risk scoring levels reflected a potential model for immune-based response status, which was essential for MSCM.

### 3.6. The landscape of tumor micro-environment immune-based cells infiltration in metastatic skin cutaneous melanoma

**Figure 7.**
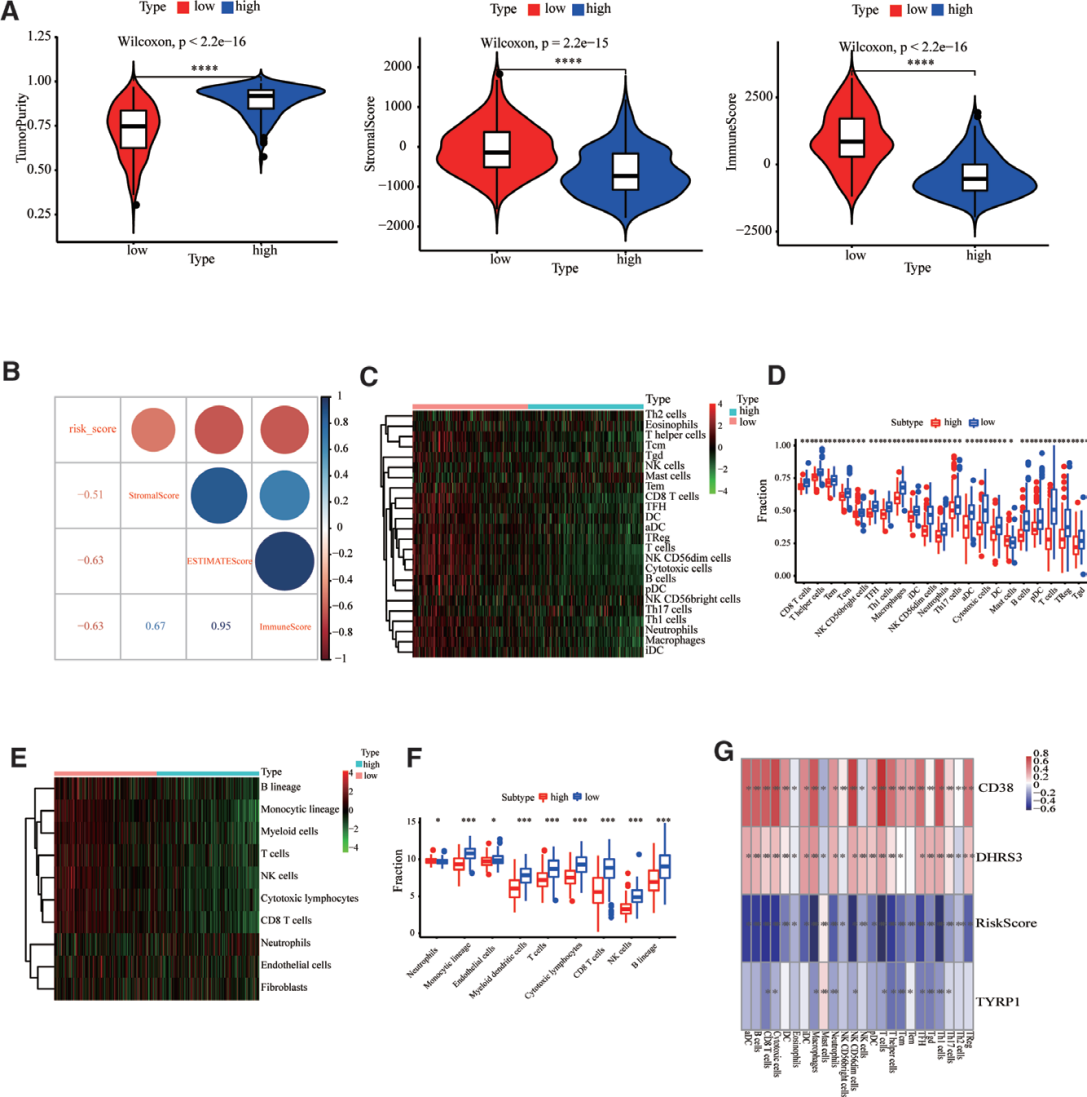
The genomic signature was linked to the composition of TME. (A) The violin plots showed the differences in tumor purity, stromal scoring, and immune-based scoring across high- and low-risk cohort cases. (B) A correlation matrix consisting of risk scoring, immune-based scoring, estimate scoring, and stromal scoring. (C, D) The cluster heat map (C) and boxplot plot (D) showed the abundance of immune-based and stromal cell populations depending upon ssGSEA analysis across high- and low-risk cohort cases. (E, F) the cluster heat map (E) and boxplot plot (F) showed the abundance of immune-based cell populations depending upon the results of MCP-counter analysis across high- and low-risk cohort cases. * indicated *P* < .05, ** indicated *P* < .01, *** indicated *P* < .001, and **** indicated *P* < .0001. TME = tumor micro-environment.

The immune-based scoring, stromal scoring, and estimate scoring were calculated for the TCGA-MSCM cohort using the ESTIMATE algorithm, which may represent the tumor microenvironment (TME). It was discovered that this prognosis-based model had a significant negative correlation with these scores (Fig. 7A and B). The abundance of 24 immune-based cell types was determined using ssGSEA in order to thoroughly characterize the landscape of TME immune-based cell infiltration in MSCM. The cluster heatmap revealed that low-risk cases were infiltrated with immune-based TME cells (Fig. 7C and D). To validate these findings, the MCP-counter method was employed to evaluate the relationship between the prognosis-based model and tumor-infiltrating immune cells. Low-risk cases exhibited a strong positive correlation with B lineage, monocytic lineage, T-cells, myeloid dendritic cells, natural killer cells, cytotoxic lymphocytes, CD8 T-cells, endothelial cells, and fibroblasts (Fig. 7E and F). In addition, Spearman correlation analyses revealed that the risk score was nearly negatively correlated with all immune-based cells (with the exception of mast cells), which exerted a robust antitumor effect (Fig. 7G). Interestingly, *CD38* and *DHR3* were positively associated with the majority of immune-based cells in the prognosis model. Combining the earlier findings, we were more certain that the 2 genes mentioned above were tumor suppressor genes (Fig. 3). It was discovered that nearly all immune-based cells were present in the low-risk cohort, indicating that an excellent prognosis may depend on the immune-based protection milieu.

### 3.7. The prognosis-based model could predict the immunotherapy

**Figure 8.**
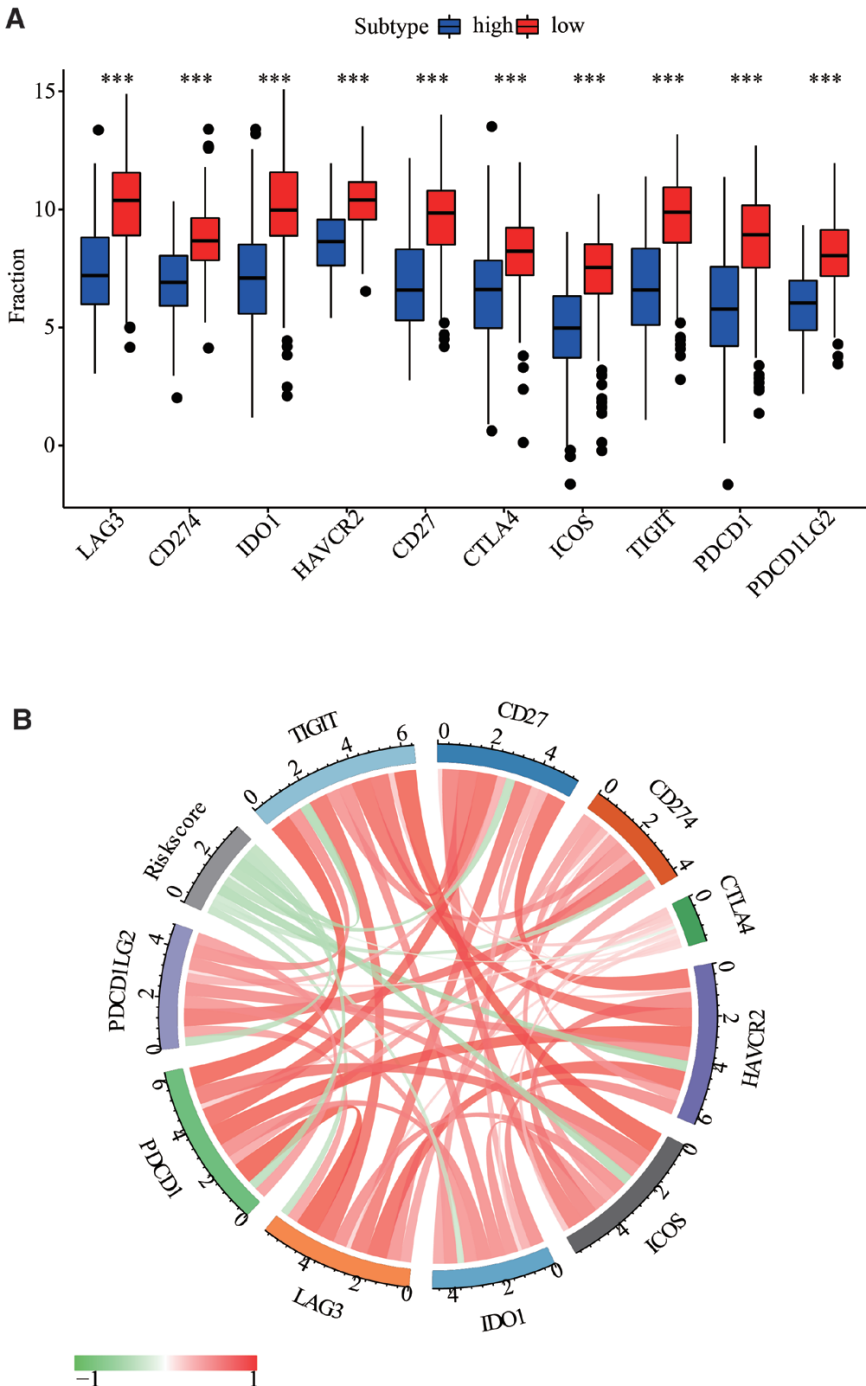
The genomic signature was linked to expression levels of 10 immune-based checkpoint genes. (A) Boxplot plot presented the expression differences of 10 immune-based checkpoint genes across high- and low-risk cohort cases. (B) the correlation chord chart demonstrated the mutual relationship between risk scoring and immune-based checkpoint genes. * indicated *P* < .05, ** indicated *P* < .01, *** indicated *P* < .001, and **** indicated *P* < .0001.

Next, we wonder if a correlation existed between the prognosis-based model and immune-based checkpoints, which may trigger the immune-based protective environment around the tumor and have been reported as predictive immunotherapy biomarkers in multiple cancers (38). Consequently, the routine immune-based checkpoints, such as *LAG3, CD274 (PD-L1*), *IDO1, HAVCR2 (TIM-3*), *CD27, CTLA- 4, ICOS, TIGIT, PDCD1 (PD-1*), and *PDCD1LG2 (PD-1LG2*), were chosen to assess the correlation with the prognosis-based model. Then, it was discovered that the expression of nearly all immune-based checkpoints was upregulated in cases with low risk (Fig. 8). These findings indicated that the genomic signature could be utilized to predict immunotherapeutic reactions.

## 4. Discussion

MSCM, a highly aggressive form of skin cancer, has attracted worldwide positive attention and become a global public concern. Although current advancements in targeted therapies like BRAF and immune-based checkpoint inhibitors have extended the survival of skin cutaneous melanoma, the prognosis of MSCM remains poor.^[6–8]^ Currently, research focuses on prognosis-based screening biomarkers for cutaneous melanoma of the skin. Using MRGs to predict the survival of skin cutaneous melanoma cases, Zeng et al developed a prognosis-based genomic signature.^[36]^ Guo et al constructed a 4-DNA methylation signature associated with the prognosis of skin cutaneous melanoma cases.^[45]^ Moreover, Hu et al established an effective risk model relating to overall survival of skin cutaneous melanoma cases based on 5-IFNγ response-linked gene.^[46]^ However, effective prognosis-based biomarkers for predicting the survival of MSCM patients are still lacking. Recent findings indicate that metabolic abnormality plays a significant role in the occurrence of MSCM and can influence the immunotherapeutic response,^[22,23]^ suggesting that MRGs may be useful biomarkers. Therefore, the relationship between MRGs and MSCM for the development of a genomic signature to predict the overall survival of MSCM cases was investigated. In addition, this study investigated the associations between the linked genomic signature of MRGs and TME.

In the present study, a 3 MRGs (CD38, DHRS3, and TYRP1) linked genomic signature was established to predict the survival of MSCM. CD38 was only regarded as a T-cell marker over the past years.^[47]^ Recent research indicates that CD38 is the predominant nicotinamide dinucleotide catabolic enzyme and can regulate a number of pathophysiological conditions, such as infection, aging, and tumorigenesis.^[48–52]^ Remarkably, CD38 has been proposed as a potential diagnostic predictor for cutaneous melanoma of the skin.^[53]^ Moreover, knockout or inhibition can suppress the metastasis of melanoma.^[54]^ This study indicated a correlation between CD38 and MSCM survival. Consequently, CD38 may play a crucial role in the development and maturation of MSCM by regulating metabolic reprogramming of nicotinamide dinucleotide and may serve as a therapeutic target. DHRS3, also known as retSDR1, is a highly conserved member of the short chain alcohol dehydrogenase/reductase superfamily and has been implicated in the retinol (vitamin A) metabolism.^[55]^ Cancer metastasis was strongly associated with DHRS3. DHRS3 was down-regulated in human triple-negative breast cancer metastases relative to normal breast tissue or primary tumors, according to research.^[56]^ DHRS3 has been reported to be negatively correlated with lymph-node metastasis in papillary thyroid cancer.^[57]^ Additionally, abnormal methylation of DHRS3 promoter CpG islands was observed in human malignant melanomas.^[58]^ Consequently, the aforementioned results were consistent with the current study’s findings, indicating that DHRS3 may serve as a biomarker for MSCM prognosis. As a prognostic marker for skin metastases, TYRP1, a member of the tyrosinase family, has been identified.^[59]^ Notably, consistent with the present study’s findings that MSCM cases with a higher level of TYRP1 expression have a poorer prognosis (Figure S1, Supplemental Digital Content, http://links.lww.com/MD/M697), another study demonstrated that TYRP1 protein is inversely correlated with survival in melanoma and that TYRP1 may refine prognosis in cases of advanced melanoma.^[60]^

Functional annotation analysis was conducted using GSVA to further investigate the biological process pathways linked to genomic signature. Interestingly, the functional annotation of high- and low-risk cohorts revealed that angiogenesis-linked pathways, which play a crucial role in tumorigenesis, and metabolism-linked pathways, etc, were predominantly enriched in the high-risk cohort (Fig. 6A and B). Furthermore, immune-based response pathways and metabolism-linked pathways were primarily enriched in the low-risk cohort (Fig. 6A and B). Notably, the risk score based on the genomic signature was strongly negative for all immune-based gene sets (Fig. 6C). Thus, the functional annotation may suggest that metabolic imbalances may activate immune-response-related pathways, resulting in a better prognosis for cases in the low-risk cohort. Meanwhile, based on the outcome of functional annotation, we conducted a thorough evaluation of the TME for both high- and low-risk cohorts. Remarkably, the high-risk cohort had higher immune-based and stromal scoring than the low-risk cohort (Fig. 7A). Moreover, both ssGSEA and MCP-counter results showed that the infiltrating levels of a large number of cells, including CD8 T-cells, macrophage, natural killer cells, Th1 cells and Th17 cells were more highly expressed in the low-risk cohort than in the high-risk cohort (Fig. 7D and E). Recent studies have shown that the cell and molecule composition of the TME can affect the immunotherapy’s effectiveness.^[61,62]^ Interestingly, the majority of immune-based checkpoints were upregulated in low-risk cases (Fig. 8), suggesting that differences in TME may influence immunotherapy response differences. As a result, 3 MRGs linked genomic signatures were highly correlated with immunotherapy response. Some promising results indicate that the CD38-linked adenosinergic pathway may be an effective target for cancer immunotherapy.^[63]^ Furthermore, CD38 could act as a receptor on the cell’s surface and played an essential role in the activation and proliferation of immune-based cells.^[54,64]^ DHRS3 was associated with M1 Macrophage and may facilitate clinical thyroid cancer treatments.^[65]^ Moreover, single-cell RNA-sequencing revealed that TYRP1 was only expressed in exhausted CD8^+^ T cell subpopulation 2 in melanoma and could serve as a potential therapeutic target for melanoma.^[66]^ Interestingly, this study also discovered that the expression of CD38, DHRS3, and TYRP1 was significantly associated with the levels of immune-based cell infiltration (Fig. 7G). All of these results suggested that MRG-linked genes (CD38, DHRS3, and TYRP1) could serve as predictors of immunotherapy’s efficacy.

## 5. Limitation

While this study has yielded important insights into the prognostic potential of metabolism-linked genes in MSCM, it is important to acknowledge its reliance on retrospective public database analyses. The inherent nature of such databases may not encompass the full variability of clinical scenarios, possibly affecting the generalizability of our findings. Additionally, the absence of protein-level validation leaves some uncertainty regarding the functional implications of the genomic signatures identified. Further validation through prospective clinical studies would be beneficial to confirm the robustness and clinical applicability of our results. These considerations gently temper the conclusions drawn and highlight the need for broader verification in future research

## 6. Conclusion

In the current study, a comprehensive bioinformatic analysis was used to establish a 3 MRGs (CD38, DHRS3, and TYRP1) linked genomic signature to predict the survival of MSCMs. Furthermore, further research suggested that CD38, DHRS3, and TYRP1 could be used to predict the efficacy of immunotherapy. These findings may have implications for the treatment of MSCM in clinical settings. However, more prospective studies will be required to validate these findings in order to provide the best targeted therapies for MSCM cases.

## Author contributions

**Data curation:** Zhongshun He.

**Funding acquisition:** Biao Xu.

**Visualization:** Biao Xu.

**Writing – review & editing:** Jing Lyu, Lechun Lyu, Xiaolin Long, Biao Xu.

## Supplementary Material











**Figure:**
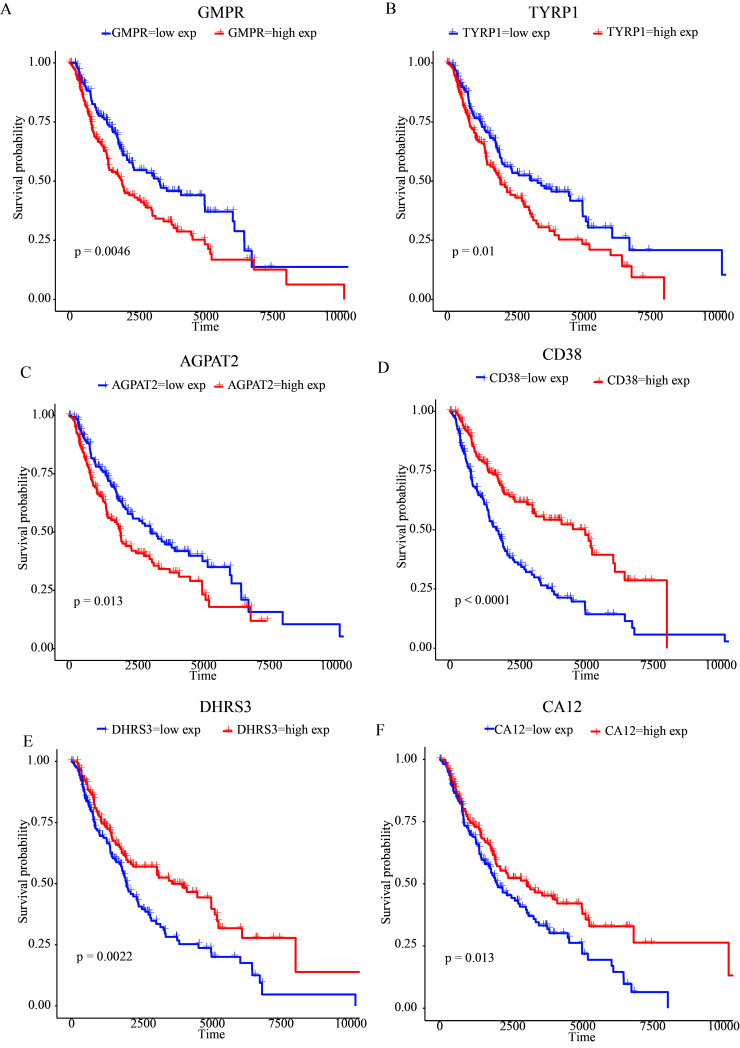




**Figure:**
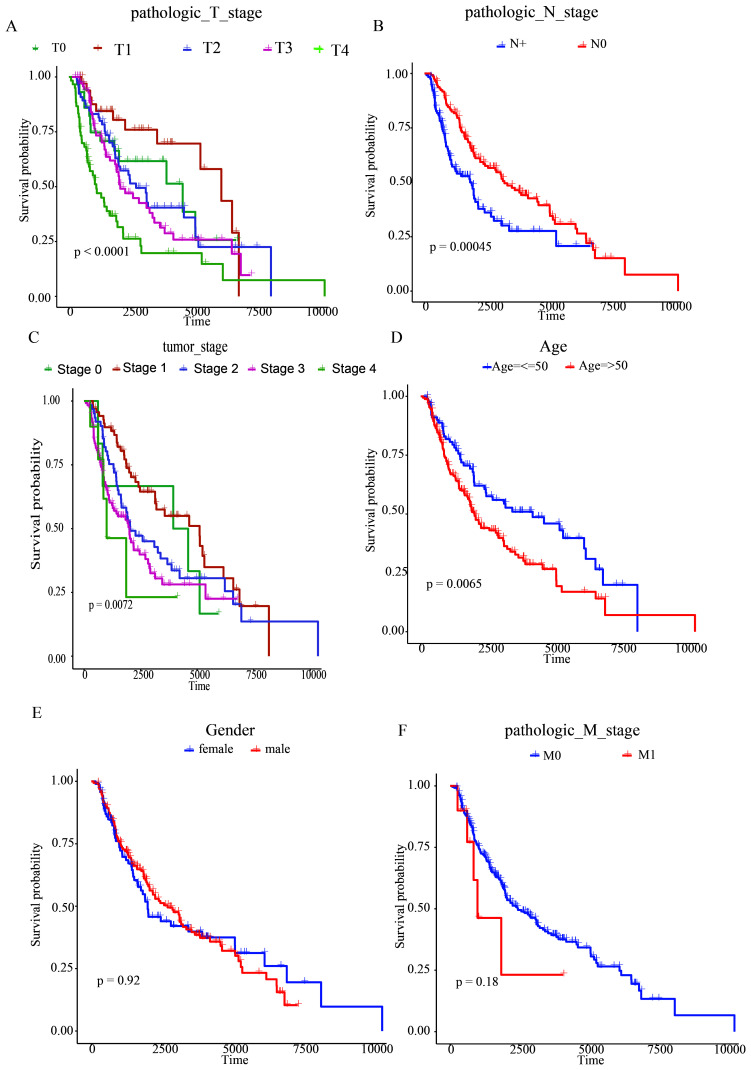


**Figure:**